# Characterizing cognitive aging of spatial and contextual memory in animal models

**DOI:** 10.3389/fnagi.2012.00012

**Published:** 2012-09-12

**Authors:** Thomas C. Foster, R. A. DeFazio, Jennifer L. Bizon

**Affiliations:** ^1^Department of Neuroscience, Evelyn F. and William L. McKnight Brain Institute, University of FloridaGainesville, FL, USA; ^2^Department of Molecular and Integrative Physiology, University of MichiganAnn Arbor, MI, USA

**Keywords:** spatial, contextual, memory, aging, hippocampus

## Abstract

Episodic memory, especially memory for contextual or spatial information, is particularly vulnerable to age-related decline in humans and animal models of aging. The continuing improvement of virtual environment technology for testing humans signifies that widely used procedures employed in the animal literature for examining spatial memory could be developed for examining age-related cognitive decline in humans. The current review examines cross species considerations for implementing these tasks and translating findings across different levels of analysis. The specificity of brain systems as well as gaps in linking human and animal laboratory models is discussed.

## Introduction

### Age-dependent decline in memory

Even in the absence of brain disorders, humans exhibit a weakening of specific cognitive processes with advancing age, including executive function, and processing speed. The most notable decline and the earliest manifestation of cognitive senescence is impaired memory. Nevertheless, not all phases or forms of memory are uniformly susceptible to aging (Albert et al., 1987; Albert, 2002; Nilsson, 2003; Hedden and Gabrieli, 2004). Aging is not associated with a serious decrement in the rate at which the elderly acquire skills, learn procedures, or form simple associations (Vakil and Agmon-Ashkenazi, 1997; Naveh-Benjamin, 2000; Peretti et al., 2002). Furthermore, aging does not have a negative impact on the long-term retention/performance of previously acquired skills and procedures (Mitchell et al., 1990; Rodrigue et al., 2005; Smith et al., 2005). Older adults experience difficulty with episodic memory (i.e., memory for recent experiences or episodes), which encompasses a specific temporal and spatial context. For example, both older and younger individuals exhibit an increase in reading speed of previously present sentences, indicating a similar level of learning of the procedural aspects of performance (Moscovitch et al., 1986). Furthermore, recognition memory was similar several hours after training, indicating that all age groups acquired the presented information. Thus, procedural memory, short term memory, and remote or semantic memories remain relatively intact. However, increased forgetting of the presented material was observed in oldest individuals when tested 2 weeks following the initial learning (Moscovitch et al., 1986). In fact, memory for contextual information (temporal order, spatial location, or source) exhibited greater impairment relative to deficits for the specific items or events to be remembered (Spencer and Raz, 1995; Old and Naveh-Benjamin, 2008). As such, diagnostic tests that specifically examine spatial memory or contextual memory (e.g., contextual fear conditioning) should be highly sensitive to aging.

### Spatial memory

Spatial memory encompasses knowledge of location within an environmental context and cognitive deficits are prominent when older adults are tested for spatial memory. Indeed, spatial learning (Weber et al., 1978; Ohta et al., 1981; Kirasic, 1991; Newman and Kaszniak, 2000) and memory (Park et al., 1982, 1983; Evans et al., 1984; Zelinski and Light, 1988; Denney et al., 1992; Uttl and Graf, 1993; de Jager et al., 2002; West et al., 2002) are particularly vulnerable to age-related decline in humans. Impaired spatial ability can have a great impact on the quality of life with advancing age, since the capacity to acquire and recall the spatial features of a novel location is critical for adaptation to the environment and the ability to live independently.

Animal models of cognitive senescence confirm that spatial memory is enormously sensitive to age. Deficits are specific to spatial memory, and like humans, little, or no age-related impairment is observed in the acquisition and retention of motor or procedural skills for primates (Walton et al., 2008) and rodents (Churchill et al., 2003; Cassel et al., 2007). For non-human primates, spatial memory appears to be one of the earliest cognitive functions to decline with age (Bachevalier et al., 1991; Rapp et al., 1997). Likewise, for rodents impaired spatial memory emerges early, in middle-age (Davis et al., 1993; Lindner, 1997; Blalock et al., 2003; Foster et al., 2003; Driscoll et al., 2006; Francia et al., 2006; Adams et al., 2008; Granholm et al., 2008; Bizon et al., 2009). The early onset and distinctive nature of cognitive decline, specific to spatial memory, suggests that specific neural systems involved in spatial navigation are exceptionally susceptible to aging.

### The hippocampus and spatial memory

The anatomical substrates for episodic and spatial memory are similar in animal models and humans. Relevant brain regions include the hippocampus and adjacent cortical areas in the temporal lobe (Nadel and Moscovitch, 1997; Nadel et al., 2000). Historically, the momentum for the idea that the hippocampus mediates memory function comes from work examining patients following surgical resection of the hippocampus to alleviate epilepsy (Scoville and Milner, 1957). The role of the hippocampus in memory consolidation was recognized in the early landmark research on the patient H.M. A role for the hippocampus in spatial memory initially arose from work describing the spatial specific discharge activity of neurons in the hippocampus (O'Keefe and Dostrovsky, 1971), and the idea that the hippocampus is critical for spatial memory was actively promoted by O'Keefe and Nadel (1978). Consistently, research has concluded that damage to the hippocampus results in impaired spatial memory in rodents (Schenk and Morris, 1985; Jarrard, 1993; Devan et al., 1996), non-human primates (Murray et al., 1989; Lavenex et al., 2006; Banta et al., 2009), and humans (Goldstein et al., 1989; Bohbot et al., 1998; Holdstock et al., 2000; Feigenbaum and Morris, 2004; Parslow et al., 2005). Indeed, the progressive decline in memory during aging is associated with a decrease in hippocampal volume in humans (Mungas et al., 2005; Kramer et al., 2007; Mueller et al., 2007; Head et al., 2008; Stoub et al., 2008; Head and Isom, 2010; Reuter-Lorenz and Park, 2010; Sexton et al., 2010), non-human primates (Picq et al., 2012), and rodents (Driscoll et al., 2006).

### Tasks for hippocampal-dependent spatial memory

A variety of mazes are commonly used for examining spatial memory in aging animals. For non-human primates, tasks that involve responding to computer screens require extensive training. In contrast, spatial information is acquired quickly in three-dimensional environments (Wang et al., 2007; Zhang et al., 2008; Haley et al., 2009). A number of spatial mazes have been developed to examine the rate of learning and duration of retention for hippocampal-dependent spatial working and reference memory in rodents, including stone maze, radial maze, Morris water maze, Barnes maze, and T-maze (Barnes, 1979; Morris et al., 1982; Beatty et al., 1985; Ingram, 1988; Bimonte et al., 2003). For this review we will focus on the acquisition and retention of novel spatial information, which remains constant across training trials, including spatial reference memory. Research on spatial working memory in well-known environments, involving the manipulation or updating of spatial information in a trial dependent manner (i.e. working memory on the radial arm maze) is addressed in more detail in the companion paper by Bizon et al., in this special issue (Bizon et al., 2012).

Tests used to examine spatial memory in humans can be categorized according to small and large scale environments. Depending on the scale of the environment, performance accuracy will depend on activity in different neural systems (Ekstrom et al., 2003; Hartley and Burgess, 2005). Small scale spatial tasks usually involve localizing objects on a two-dimensional field and performance is associated with activity in the parietal lobes (Kosslyn and Thompson, 2003). Memory deficits in small scale environments are not as robust as in large scale environments and older individuals may show little or no impairment when distinctive spatial cues are used (Cherry and Park, 1993) or short retention intervals are employed (Olson et al., 2004). A consistent age-related deficit is observed for spatial memory tested in large-scale environments (Weber et al., 1978; Simon et al., 1992; Uttl and Graf, 1993; Wilkniss et al., 1997; Newman and Kaszniak, 2000). Early studies, using slide shows to simulate a walk through large-scale environments, demonstrated robust spatial memory impairments in older individuals (Ohta et al., 1981; Hunt and Roll, 1987; Kirasic, 1989; Kirasic and Bernicki, 1990; Kirasic et al., 1992; Lipman and Caplan, 1992). More recently, computer-based virtual environments have been employed to show age-related deficits specific for processing the relationship between multiple environmental cues (Moffat et al., 2001, 2007; Moffat and Resnick, 2002; Iaria et al., 2009; Head and Isom, 2010; Jansen et al., 2010; Plancher et al., 2010). The computer generated environments permit control of cue location and task complexity and the use of computer controls can compensate for physical disability. Virtual environments are more ecologically valid than small scale tests and can be programmed to mimic spatial tasks used to characterize age-related impairments in animal models.

### Example of rodent water maze protocols

The Morris water maze is the gold standard for examining altered spatial memory in relation to hippocampal damage or associated with aging in rodents, and versions of the maze have been adapted for examining aging in dogs (Salvin et al., 2011) and humans (Newman and Kaszniak, 2000; Moffat and Resnick, 2002; Driscoll et al., 2003; Fitting et al., 2007; Antonova et al., 2009). There is no set guideline as to what materials and pool sizes should be used (Guidi and Foster, 2012). Depending on the animal color, paint, or dye may be added to the water to provide the visual contrast need for computer tracking programs. Moreover, the animal model (rat or mouse) should be considered when deciding on pool size and escape platform size. In general, pool sizes range from approximately 1.7–1.8 m for rats and 1–1.5 m for the mice and platform diameters are ~12–15 cm for rats and ~10–11 cm for mice. Too large a pool or too small a platform will result in slower learning and possible exhaustion due to extensive swimming.

**Figure 1:**
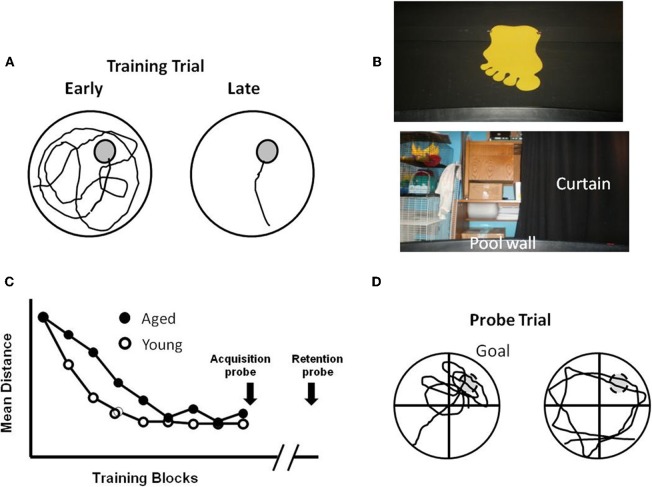
**Performance differences during aging for training on the water maze. (A)** Schematic illustration of the pool, with an escape platform (gray circle) in the upper right hand quadrant. The line indicates the path length to find the platform, which decreases from the earlier to later training trials. **(B)** Spatial cues include posters attached to the room walls, equipment within the room, and lights. In addition, a large black curtain can be pulled around the pool to limit spatial cues. **(C)** Performance curves for young and aged animals illustrating similar performance on the first trial block. Young animals quickly learn the location of the platform, reducing the distance traveled to escape. Aging is associated with a slower rate of learning. Probe trials can be delivered at the end of training to confirm the acquisition of a spatial search strategy, or after a delay (e.g., 24 h) to determine retention. **(D)** During a probe trial, the platform is removed and the search pattern is focused on the area (i.e., goal quadrant) in which the platform was located. Search behavior focused on the goal quadrant (Left) indicates acquisition or retention of a spatial search strategy. Animals that fail to learn a spatial search strategy may employ an egocentric search strategy, swimming at a distance from the pool wall that approximates the distance of the platform from the wall.

A cue discrimination version of the task is employed in order to specify that behavioral differences are not due to age-related sensory-motor or motivational deficits. For the cued discrimination task, a floor to ceiling curtain surrounds the pool in order to limit spatial cues (Figure 1B). The escape platform is highlighted by attaching a flag to the platform and the platform is raised above the level of the water in the pool. In the cued task, the platform is placed in different positions for each trial and the subject is trained to locate the visible platform. The platform should be located away from the wall to prevent the animal from finding the platform by circling next to the wall (i.e., thigmotaxis), and discouraging animals on the platform from leaping out of the pool. Animals that cannot locate the visible platform are removed from further consideration. In addition, training on the cue discrimination task ensures that animals learn the procedural aspects of the task, including how to swim and the fact that the pool wall is not a route of escape (Vorhees and Williams, 2006). Animals that complete the cue discrimination task can see, swim, are motivated to escape from the pool, and have the basic skills and knowledge needed to perform the task. These animals are ready for testing of acquisition, retention, and flexible use of spatial information.

For the spatial version of the task, the curtains are removed and mice or rats are trained to find an escape platform in a pool of water using spatial cues in the room (Figure 1). The procedure by which rodents are tested in the spatial water maze varies greatly and consists of multiple versions. In general, the platform is hidden below the water level and remains in the same spatial position throughout training. With each trial, the rodent is gently placed in the pool at a randomized starting position. The number of training trials can vary between one or more each day for several days (Figure 3) or training is conducted in a single day and trials are arranged in training blocks, containing several training trials per block (Figure 1C). Learning is observed as a decrease in the path length during training as the animals traverse the environment to escape from the pool. In addition, probe trials are employed immediately after training to test the extent of learning and at various delays following training in order to examine retention. For probe trials, the escape platform is removed and the animal's swim pattern is recorded (Figure 1D). Animals that learn and remember the spatial location of the platform focus their search on the location that previously held the platform.

### Virtual environments for testing humans

**Figure 2:**
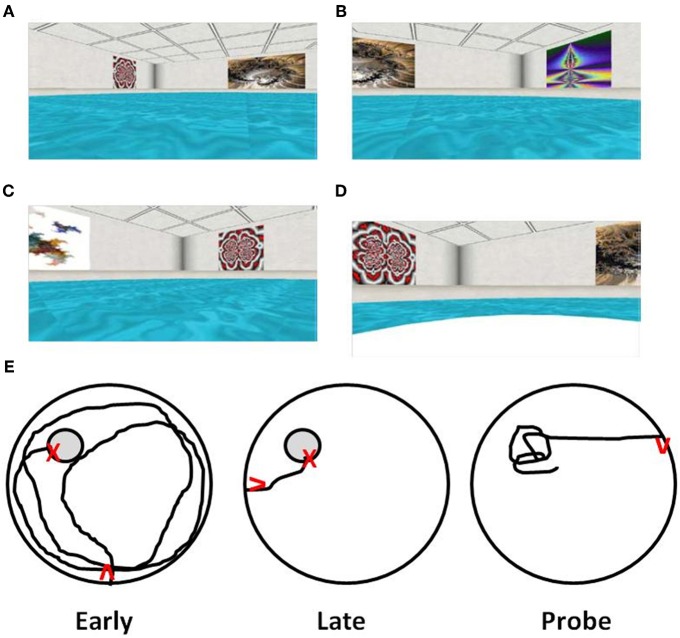
**Computer-graphic ARENA example views as seen by participants during “Hidden Target Trials” (Jacobs et al., 1997; Springer, 2010).** The views include the **(A)** northwest quadrant, **(B)** northeast quadrant, **(C)** southwest quadrant, and **(D)** a view of the northwest quadrant containing the target, shown after arrival at the target. **(E)** Examples of path length to find the hidden target (gray circle) during the first training trial of acquisition training (Early), direct path to target of later training trial indicating learning (Late), and a probe trial with path focused on the location that had contained the target (Probe). Red arrows indicate start position/orientation) and red X's indicate the end point of navigation.

Virtual environments, including virtual versions of the water maze (Figure 2), have been used to examine hippocampal function in amnesic humans. In young adults, the behavior is similar to that observed for young rats. The path length to find the hidden target decreases with training. In addition, probe trials indicate that search behavior is focused on the location of the target (Figure 2E). Research confirms that damage to the hippocampus results in impaired spatial navigation (Skelton et al., 2000; Astur et al., 2002; Bohbot et al., 2004; Bartsch et al., 2010; Goodrich-Hunsaker et al., 2010). Recent studies have examined spatial learning and memory during aging in virtual reality environments and mazes (Iaria et al., 2009; Head and Isom, 2010; Plancher et al., 2010). Analogous to aged rats and mice, elderly individuals exhibit deficits involving increased path length during acquisition and impaired performance on probe trials (Moffat et al., 2001, 2007; Moffat and Resnick, 2002; Driscoll et al., 2003). Importantly, while all age groups exhibit acquisition on a virtual environment maze, middle-aged (40–60 years), and aged subjects (>60 years) were slower to learn and exhibited increased spatial memory errors (Thomas et al., 1999; Moffat et al., 2001; Moffat and Resnick, 2002; Driscoll et al., 2005; Jansen et al., 2010), indicating that the tasks are sensitive to the early emergence of cognitive decline. Finally, performance in virtual environments correlates with other hippocampus-sensitive tasks including measures of episodic memory and spatial ability (Moffat et al., 2001; Driscoll et al., 2005).

The results for studies that employ virtual environments confirm that aging is associated with impaired contextual memory; including recall of spatial information and that tasks involving spatial navigation through virtual environments are sensitive for the early detection of cognitive deficits. The continuing improvement of virtual environment technology for testing humans signifies that widely used procedures employed in the animal literature could be developed for examining age-related cognitive decline in humans. The next section examines aspects of testing procedures and methods that are important in cross species testing.

### Cross species procedure considerations

Several reviews have discussed the proper procedures for employing spatial swim mazes for rodents (Brandeis et al., 1989; Vorhees and Williams, 2006) and the factors that must be considered when examining aged animals (Foster, 1999; van der Staay, 2002). The work highlights attributes of the task and of aging that could confound behavioral measures of spatial learning and memory, including sensory-motor deficits, fatigue, and stress.

### Sensory-motor function

It is important to keep in mind that virtual environments do not involve true translocation through space. Perceptions are different and virtual environments can cause disorientation and nausea in some subjects (Regan and Price, 1994; Howarth and Finch, 1999; Moffat et al., 2001). Furthermore, motor activity associated with moving through a virtual environment (i.e., a joystick) is different than walking or swimming. Voluntary motor activity provides important information that influences spatial cognition. For example, learning and memory are increased when subjects actively, rather than passively explore the environment (Sauzeon et al., 2011). There may be an interaction between walking on a treadmill and the virtual experience of walking on stress responsiveness (Plante et al., 2003). Finally, the addition of a motor component to the virtual environment, e.g., treadmill walking, improved spatial learning in older adults; however, the motor component did not eliminate deficits in spatial memory (Lovden et al., 2005), suggesting the possible dissociation of impairments in learning and memory processes.

For rodent studies, deprivation of food or water or exposure to aversive stimuli is employed to motivate acquisition. However, there is concern that stress or the level of motivation associated with food deprivation or shock may not be equivalent across age groups (Gage et al., 1984). Rodents are excellent swimmers and they are inherently motivated to escape water. However, motivation is decreased as water temperatures approach body temperature (Wever, 1932) and cold temperatures (i.e., below room temperature) can be stressful, particularly for aged animals (Lindner and Gribkoff, 1991; Mabry et al., 1996). Stress can be reduced by drying and warming the animals between trial blocks and habituation procedures, including cue discrimination training.

For hippocampal-dependent spatial behavior, learning is based on the relationship of cues outside the pool. Therefore, animals must be able to detect extramaze cues. The nature of extramaze cues employed in water maze and exactly what constitutes a sufficient number of cues is not entirely clear. Relative to humans, rodents have poor visual acuity; however, using only one or two highly salient cues may promote cue-response learning over flexible spatial learning (Gerlai et al., 2002). In humans the salience of spatial cues, including color and familiarity facilitate spatial learning (Ruddle et al., 1997; Barkas et al., 2010). For rodents, simplification of cues (large shapes affixed to a curtain or wall surrounding the maze) tends to improve place learning relative to complex patterns (Burwell et al., 2004). Indeed, there is evidence that differences in the spatial environments, with respect to the complexity of visual cues, can differentially engage extrahippocampal structures. Perirhinal and postrhinal cortices confer the primary sensory association information from neocortex to hippocampus directly and via entorhinal cortex. Lesion studies of perirhinal and postrhinal cortices are somewhat contradictory regarding the contribution of these areas to spatial water maze performance (Aggleton et al., 2000). While intact performance has been reported in some cases (Bussey et al., 1999; Burwell et al., 2004), others find such lesions can markedly impair spatial learning and subsequent probe trial retention (Kaut and Bunsey, 2001; Liu and Bilkey, 2001). One factor that may account for these differences concerns the complexity of the maze environment. Lesions appear to spare performance in instances in which extramaze cues are discrete and simple, suggesting that under these conditions, information from polymodal associational regions may not enhance or prove beneficial for subjects to learn and remember a spatial location. However, in cases in which the water maze environment is more complex, such higher order cortical regions may be more heavily recruited and more essential to successful learning of the platform location. As such, spatial learning performed within the framework of a complex spatial environment with more varied cues (e.g., an open room containing laboratory furniture and other objects) could slow acquisition of a spatial location.

### Previous experience

In addition to identifying animals with sensory-motor deficits, training on the cue task can reduce stress associated with training (Mabry et al., 1996) and ensure that animals learn the procedural skills needed to perform the task (Vorhees and Williams, 2006). Consequently, animals may perform better on the spatial version of the task when they are first trained on the cue task. When animals are initially trained on the spatial version of the task, performance differences may result from impaired acquisition of the procedural aspects of the task (Gerlai, 2001). Indeed, when spatial training is examined prior to visual platform training (Rapp et al., 1987), no age difference is observed for distance on subsequent visible platform training, suggesting that elderly individuals acquired the procedural skills needed to match young individuals while performing the spatial task. A correspondence between performance on the spatial task and cue discrimination task may indicate more global impairments and has been used as an indication of the extent of pathology in mouse models of neurodegenerative disease (Arendash and King, 2002; Leighty et al., 2004).

Virtual environments do not normally induce a significant stress response in humans (Bullinger et al., 2005; Driscoll et al., 2005). However, previous experience with computers and practice in navigating virtual environments are important variables that must be controlled. Compared to younger subjects, older individuals usually have less experience with computers and joystick controls, which can influence performance on virtual mazes (Moffat et al., 2001; Carelli et al., 2011). This differential experience likely contributes to a marked age-related impairment in performance for the first training trial (Moffat et al., 2001; Driscoll et al., 2005). These differences may diminish on subsequent trials as older individuals become familiar with the controls. Indeed, when spatial training is examined first, no age difference is observed for distance on subsequent visible platform training (Moffat and Resnick, 2002; Driscoll et al., 2003), suggesting that, during performance of the spatial task, elderly individuals acquired the procedural skills needed to match younger individuals. Importantly, when experience with computers and joystick controls is taken into consideration, age effects on spatial memory for a virtual environment continue to be apparent (Moffat et al., 2001; Driscoll et al., 2003; Jansen et al., 2010).

### Sex differences

Sexually dimorphic use of spatial strategies have been noted in rodents (Galea et al., 1996; Frick et al., 2000; Veng et al., 2003; Kanit et al., 2005; Benice et al., 2006) and humans (Astur et al., 1998; Driscoll et al., 2005; Rizk-Jackson et al., 2006). Sex differences may be magnified during aging due to differences in activation of the hippocampus, gene transcription, hormonal influences, or susceptibility to oxidative stress and inflammation (McEwen et al., 1997; Gron et al., 2000; Berchtold et al., 2008; Talboom et al., 2008). In particular, there is considerable interest in the role of sex steroids and their receptors in regulating hippocampal function and the etiology of age-related cognitive decline (Conrad and Bimonte-Nelson, 2010; Daniel and Bohacek, 2010; Foster, 2012b).

### Massed and distributed training

Like humans, aging rodents exhibit a progressive decline in cognitive function and wide-ranging heterogeneity in the extent of memory impairment. A role for the hippocampus in the rapid acquisition of episodic memories and slower associative learning has been suggested (Meeter et al., 2005) and these functions may exhibit differential aging (Foster, 2012a). Training schedules can be designed to focus on these processes determining the sensitivity of the Morris escape task for detection of the emergence of acquisition and retention deficits in middle-age rodents (Foster, 2012a). For example, the hippocampus is involved in the rapid acquisition, consolidation, and recall of spatial information (Lee and Kesner, 2004; Nakazawa et al., 2004), processes that are interdependent, but potentially dissociable. Repeated acquisition training is designed to specifically examine rapid flexible spatial learning. For this task, the goal position is changed across sessions such that the animal must rapidly learn novel escape locations. Repeated acquisition training is sensitive to the emergence of cognitive decline and, for most rat species, can detect deficits by 18 months of age (Foster, 2012a).

In addition to impaired rapid acquisition of trial specific spatial information, delay-dependent effects associated with impaired retention are a consistent finding during aging (Foster, 1999). The sensitivity of behavioral tasks for detecting memory deficits can be enhanced by using a single training session (Lal et al., 1973; Vasquez et al., 1983). The retention of information following massed training is not as strong as memory acquired following distributed training (Dash et al., 2002; Spreng et al., 2002; Commins et al., 2003). Furthermore, the rapid acquisition of spatial memories is highly sensitive to hippocampal function (Martin and Clark, 2007). When training is massed into a single session, aged animals may exhibit slower learning during training (Figure 1C). It is important to distinguish between the use of a hippocampal-dependent allocentric or spatial strategy from an egocentric or cue-response strategy that depends on extrahippocampal regions. The acquisition of a spatial search strategy following a single training session can be confirmed via probe trial, by calculating the portion of time spent searching in the quadrant that originally held the escape platform (Figure 1D). In contrast, animals may use a cue-response strategy, swimming at a specified distance from the pool wall in order to locate the platform. Once it is clear that an animal has acquired a spatial search strategy, a subsequent probe trial may be delivered at more distant time points in order to evaluate retention. Middle-age animals exhibit memory deficits examined 24 h after acquisition and the probability of memory impairment increases with advancing age (Aitken and Meaney, 1989; Foster et al., 1991; Mabry et al., 1996; Foster, 1999; Norris and Foster, 1999; Blalock et al., 2003; Foster et al., 2003; Driscoll et al., 2006; Foster and Kumar, 2007).

**Figure 3:**
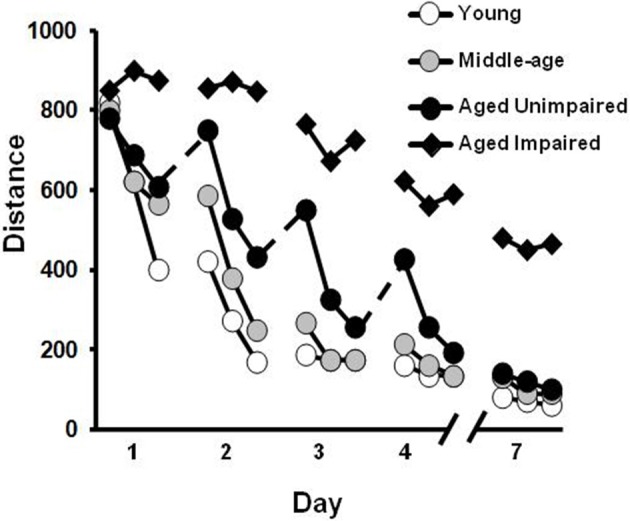
**Differences in acquisition of a spatial reference memory during training distributed across several days.** The graph illustrates the distance to find the hidden platform during training trials each day for middle-aged (gray circles), and young (open circles) animals. Older animals may exhibit an initial saw-tooth pattern of performance across days (dashed lines) suggesting increased forgetting. However, this group is able to acquire a spatial reference memory over the course of training and is, therefore, classified as aged unimpaired (filled circles). In contrast, a subgroup of aged animals may exhibit profound learning deficits such that they are unable to acquire a spatial reference memory with repeated training to the same location (filled triangles).

When training is distributed across several days, an initial age-related difference may be prominent for the first trial on each day, suggesting forgetting across days of training (Gage et al., 1984; Rapp et al., 1987; Aitken and Meaney, 1989; Foster, 2012a). However, many older animals can acquire a spatial reference memory with continued training. In addition, several labs have characterized a subgroup of aged rats that continue to exhibit impaired acquisition of a spatial reference memory following multiple days of training (Figure 3). For impaired animals, the deficits are reliable, particularly when training consists of only one to two trials per day (Gallagher et al., 1993; Ivy et al., 1994; Frick et al., 1995; Yamazaki et al., 1995; Mabry et al., 1996; Lindner, 1997; Rowe et al., 1998; Colombo and Gallagher, 2002; Schulz et al., 2002; Collier et al., 2004; Bizon et al., 2009; Bergado et al., 2011). Interestingly, middle-age animals that exhibited impairments on the repeated acquisition version of the task, could still acquire a spatial reference memory with training distributed across several days (Miyagawa et al., 1998; Nyffeler et al., 2010). The discrepancy between impaired rapid flexible spatial learning and intact trial-independent knowledge is consistent with differential aging of diverse memory systems (Salmaso, 1993; Burke and Mackay, 1997; Spaan et al., 2003) or mechanisms (Foster, 2012a), and suggests that impairment of memory for trial-independent information represents a later phase of decline associated with greater cognitive dysfunction. In this regard, it is important to point out that reliable deficits in the ability to incrementally acquire trial-independent spatial knowledge becomes prominent in a subset of animals as they reach ~90% of their average life span (LaSarge and Nicolle, 2009) and may be indicative of a progressive aspect of age-related cognitive decline (Gallagher and Burwell, 1989; Zyzak et al., 1995; Rowe et al., 1998; Frick et al., 2000; Schulz et al., 2002; Collier et al., 2004; LaSarge et al., 2007; Matzel et al., 2008; Bergado et al., 2011).

Mouse models of aging have advantages for examining molecular mechanisms of learning, memory, and aging due to the ability to induce genetic modifications. Previous studies on age-related changes in spatial memory on the water maze have generally employed training distributed across several days. However, it is becoming apparent that massed training schedules make use of distinct molecular memory mechanisms that are sensitive to aging (Foster et al., 2001; Malleret et al., 2001; Genoux et al., 2002) and distributed training procedures can mask memory deficits in mice with mutations of genes linked to memory. Therefore, researchers have developed versions of the water maze for mice, that focus on rapid acquisition in order to characterize more subtle changes associated with aging, age-related disease, the function of hippocampal subregions, and molecular mechanisms of memory (Kogan et al., 1997; Jones et al., 2001; Genoux et al., 2002; Magnusson et al., 2003; Vaillend et al., 2004; Nakashiba et al., 2008; Gulinello et al., 2009; Malleret et al., 2010).

For studies using virtual environments, including those that mimic the water maze, age-related deficits are apparent following a single training session. Moreover, a single training session is sensitive to the emergence of impaired cognition, such that a decrement in spatial learning and memory can be observed in middle-aged humans (Thomas et al., 1999; Driscoll et al., 2005; Jansen et al., 2010). The use of single training sessions also has the advantage of limiting subject dropout that could occur when multiple days of testing are employed.

Similar to animal studies, for aged human subjects, the initial age-related difference in acquiring spatial information on a virtual maze is diminished with continued training (Jansen et al., 2010). The initial learning and asymptotic performance phases may depend on the use of allocentric and egocentric strategies, respectively, and involve different brain systems (Hartley and Burgess, 2005; Bohbot et al., 2007; Etchamendy and Bohbot, 2007; Etchamendy et al., 2011). The results indicate that a single training session is more likely to engage hippocampal systems and is sensitive to the early detection of age-related deficits in the acquisition of spatial information in humans and animal models.

### Verbal instruction and reports

The use of similar tasks that do not rely on language may permit examination of similar experimental factors across species. However, for humans some verbal instruction is typically provided, usually to elucidate the goal of the task. Such instruction could influence strategy selection and learning rates. On the other hand, post-test debriefing can also be employed to better understand the cognitive processes engaged during task performance. Interestingly, impaired verbal recall of environmental cues may differentiate normal aging from Alzheimer's disease (Cushman et al., 2008; Widmann et al., 2012).

### Translating findings across different levels of analysis: physiological mechanisms

Rodents offer several benefits as models for investigation of the mechanisms and potential treatment of age-related cognitive decline. Similar to humans, information that requires hippocampal processing is particularly vulnerable to age. Importantly, rodents are not subject to neurodegenerative diseases such as Alzheimer's disease, suggesting that cognitive decline is due to aging of physiological processes rather than cell death (Rapp and Amaral, 1992; Baxter and Gallagher, 1996; Foster, 2006). Finally, as noted above, like humans, rodents exhibit wide-ranging heterogeneity in the extent of memory decline. This variability can be used to better define the progression of cognitive senescence and investigate age-related changes in biological mechanisms critical to rapid acquisition, consolidation, or recall of spatial information (Foster, 2012a).

Considerable research has identified biological markers of age-related spatial memory impairments in rodents (Foster, 1999, 2006; Rosenzweig and Barnes, 2003; Wilson et al., 2006; Burke and Barnes, 2011; Small et al., 2011). For example, older animals exhibit reduced excitability of hippocampal neurons, a decrease in hippocampal synaptic transmission, and impaired NMDA receptor dependent synaptic plasticity processes that are thought to underlie memory (Foster, 2007). In addition, the stability of spatial specific discharge activity of hippocampal cells declines during aging, possibly due to impaired NMDA receptor dependent synaptic plasticity (Barnes et al., 1997; Kentros et al., 1998; Guzowski et al., 1999). Due to the invasive nature of research to examine these mechanisms for memory function, studies that directly examine physiological changes that cause memory decline cannot be performed in humans. However, several studies have employed analogous spatial tasks across species and examined hippocampal activity in relation to spatial memory. For example, treatment with NMDA receptor antagonists disrupts performance on the water maze in rodents (Bannerman et al., 1995), spatial memory in monkeys (Wang et al., 2007), and virtual water maze performance in humans (Rowland et al., 2005). The impairment mimics the decline in rapid acquisition and impaired intermediate term spatial memory observed during aging (Foster, 2012a).

Studies that employ local injections of drugs to reversibly inactivate the hippocampus indicate that blockade of hippocampal activity impairs the formation and recall of spatial memory (Clark et al., 2005; Broadbent et al., 2006, 2010; Loureiro et al., 2012). In elderly humans, impaired retrieval of spatial contextual information is associated with decreased hippocampal activity during the encoding phase (Moffat et al., 2006; Antonova et al., 2009; Kukolja et al., 2009). The shift in activity could represent an impaired ability to activate the hippocampus or differences in ongoing cognitive processes. Selection of an allocentric or egocentric (i.e., spatial vs. cued) response strategy is associated with increased activity in the hippocampus and caudate, respectively (Bohbot et al., 2004; Etchamendy and Bohbot, 2007; Banner et al., 2011). In contrast to younger subjects, during the initial exposure to a novel environment, older humans and rodents tend to decrease use of a hippocampal-mediated allocentric response strategy in favor of egocentric (i.e., cued) strategies involving the caudate nucleus (Barnes et al., 1980; Rodgers et al., 2010; Banner et al., 2011; Kumar et al., 2012).

Hippocampal activity is modulated by voluntary movements (Oddie and Bland, 1998). In rodents, passive transport through an environment is more likely to engage caudate-dependent egocentric learning (Abraham et al., 1993) and reduce the spatial selective responsive activity of hippocampal cells (Foster et al., 1989; Abraham et al., 1993; Ono and Nishijo, 1999). Likewise, active exploration of the virtual environment is associated with increased hippocampal activity and better spatial learning in humans (Sauzeon et al., 2011). The relationship between hippocampal activity and spatial memory indicates that the increase in hippocampal activity during voluntary exploration contributes to memory differences associated with active and passive learning (Voss et al., 2011).

Exercise is a relatively non-invasive treatment which has been suggested to rejuvenate the hippocampus of rodents, by increasing cell excitability and restoring synaptic plasticity (Kumar et al., 2012). Despite evidence that exercise revitalizes hippocampal physiology, the effects of exercise on spatial memory are debated (Colcombe and Kramer, 2003). In humans, a 6-month exercise intervention initiated in middle-age increased hippocampal activity during encoding of a virtual environment (Holzschneider et al., 2012). However, spatial learning was not different between exercise and non-exercise groups. The results suggest that more research will be needed in order to determine how exercise influences hippocampal aging.

### Contextual fear conditioning

Conditioned fear employs classical conditioning to associate a *context* and a *cue* with a brief, distressful stimulus [the unconditioned stimulus (US)]. In animal studies, the context is established using an isolation chamber with static visual, olfactory, auditory and tactile elements. In human studies, the context can be established using carefully prepared rooms or a virtual reality apparatus. The cue is a transient stimulus: a tone, light, odor, or neutral image activated prior to the aversive stimulus. The cue is referred to as the conditioned stimulus (CS). In animal studies, the US is usually a brief foot shock or eye puff, while in human studies aversive imagery, electrical shock, or an eye puff have been employed. Fear can be assessed behaviorally [i.e., freezing behavior in rodents (Blanchard and Blanchard, 1969)] or physiologically [e.g., skin conductance response (SCR), heart rate, e.g., (Alvarez et al., 2008)]. In humans, fear has also been assessed via direct interview or brief standardized psychological tools [State-Trait Anxiety Inventory, STAI-S, or Subjective Units of Distress scale, SUD, e.g., (Fredrikson et al., 1995)]. Table 1 lists the basic elements of fear conditioning for rodent and human studies.

**Table 1:** **Basic parameters for fear conditioning in rodents and humans**.

**Context**	**Conditioned stimulus**	**Unconditioned stimulus**	**Response**
**RODENT**			
Modular isolation chamber	Tone	Foot shock	Freezing behavior
– Static visual stimuli	Light pulse	Eye puff	– Video analysis
– Changeable floors/walls			– Infrared beam crossing
– Static odor			– Load cell platform
– Background noise			Physiological
– Chamber light level			– Heart rate
			– Blood pressure
			– Subcutaneous response
**HUMAN**			
Specifically prepared rooms	Tone	Electrical shock	Physiological
Virtual reality	Light pulse	Eye puff	– Heart rate
	Neutral faces/images	Aversive images	– Blood pressure
	Transient odor		– Subcutaneous response
			Electromyography
			Psychological tools
			– State-Trait Anxiety Inventory
			– Subjective Units of Distress

**Figure 4:**
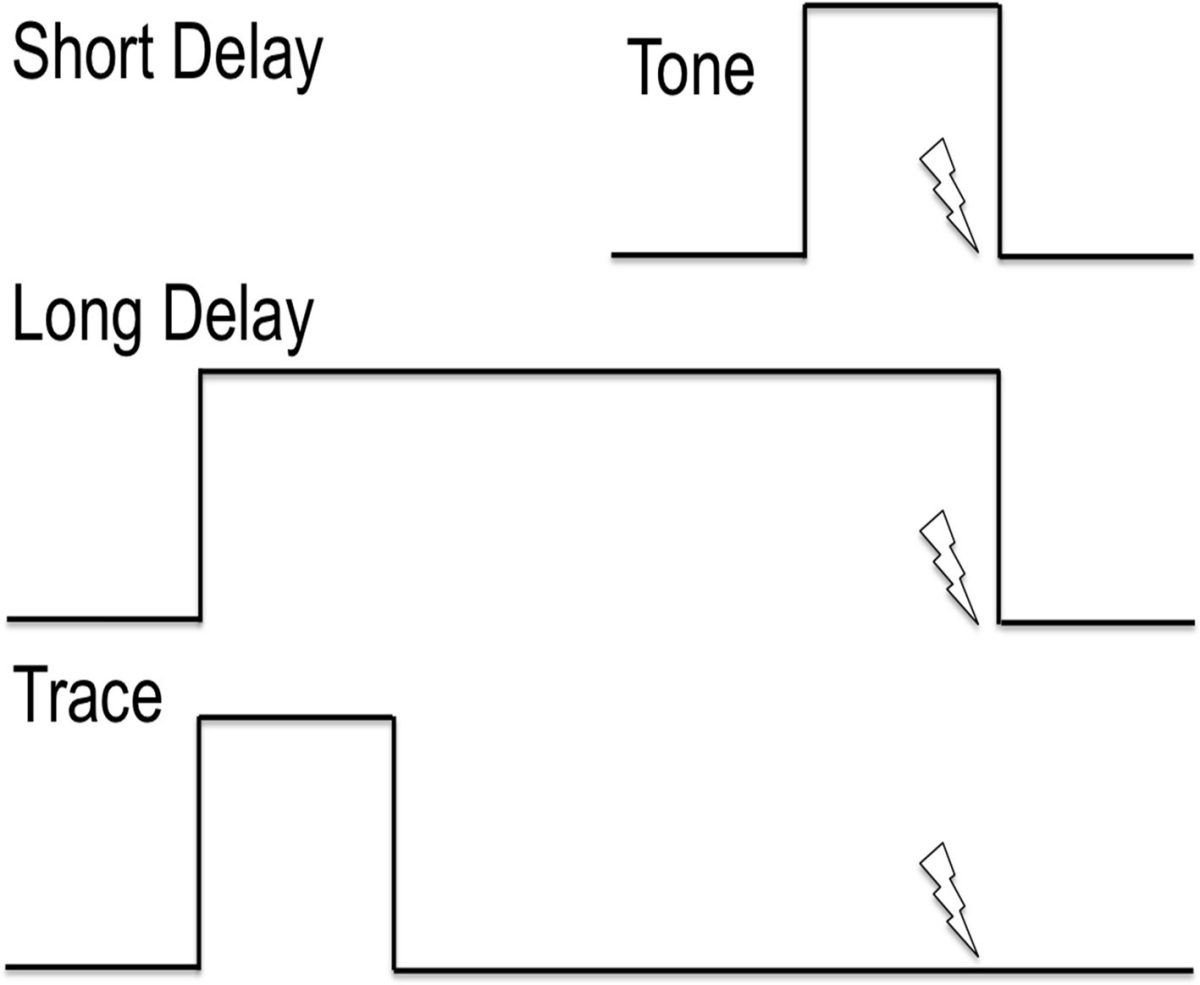
**Explanation of cued delay conditioning vs. cued trace conditioning.** The lines indicate the timing of the tone as conditioned stimulus. The lightning bolt indicates the timing of the foot shock or other aversive unconditioned stimulus. Delay conditioning is independent of the hippocampus, while trace conditioning requires hippocampal activity.

It is important to note that the stable elements of the environment contribute to the context, while a transient element (such as a brief tone preceding the US) serves as the cue. The temporal contiguity of the cue relative to the US is a crucial experimental factor. In *delay conditioning*, the US is delivered at the end of the CS. For example, a 30 s tone (CS) is played and the aversive electrical shock (US) is delivered *during* the last second of the CS (Figure 4). *Trace conditioning* refers to the introduction of a brief temporal interval between the end of the CS and the onset of the US. In this case, an interval of 0.5–10 s separates the end of the CS and the onset of the US (Figure 4).

## The neuroanatomy of contextual fear conditioning

### Rodent studies

Traditionally, the hippocampus is selectively required for contextual fear conditioning, while the amygdala plays a role in both contextual and delayed cued fear conditioning (Selden et al., 1991; Kim and Fanselow, 1992; Phillips and LeDoux, 1992). When trace conditioning is employed, cued learning and memory is also dependent on the hippocampus due to the memory requirement of “storing” the CS until the US occurs (e.g., McEchron and Disterhoft, 1999). Here we will emphasize contextual fear, given the focus of this section on the spatial memory component of the context.

The behavioral procedures required for contextual fear conditioning vary widely across laboratories, contributing to apparently contradictory results related to the role of the hippocampus. For example, Maren et al. demonstrated that lesions of the dorsal hippocampus 1 week before conditioning failed to interfere with either the acquisition or expression of contextual fear (Maren et al., 1997). In direct contradiction to the traditional valuation of conditioned fear, this group also reported that hippocampal damage 1 week prior to training resulted in deficits in the cued CS using the delayed conditioning paradigm. In this study, three tone-shock pairings (10 s, 2 kHz tone; 2 s, 1 mA shock) were employed. To assess CS-US fear expression, the CS was played during the entire 8 min test period to equate the durations of the context and cue retention tests.

As noted above in the previous sections on spatial learning, the sensitivity of behavioral tasks in detecting hippocampal-dependent memory deficits is enhanced by using a single training event. When a single conditioning trial is employed (i.e., one shock in context), acquisition of contextual fear is indeed impaired by pre-trial dorsal hippocampal lesions (Wiltgen et al., 2006). Taken together these studies demonstrate that the role of hippocampus in the acquisition of contextual fear depends critically on experimental details, specifically increasing the number of training sessions can overcome functional deficits of the hippocampus. There are also possible interactions between the context and CS, employed in the combined paradigm used in the Maren et al. (1997) study. The Wiltgen et al. (2006) study used a pure contextual conditioning paradigm (no CS-US pairing). The essential point is that pre-training hippocampal damage does not necessarily impair fear conditioning. The adult mammalian brain exhibits a remarkable capacity to adapt to functional deficits and brain injury. As such, in healthy adult rodents, other brain regions can compensate for hippocampal deficits depending on the experimental parameters.

The ventral hippocampus also plays a role in contextual fear conditioning. In rats, lesions of the ventral hippocampus generated contextual fear deficits equivalent to complete hippocampal lesions, even when a strong conditioning paradigm is employed (10 tone-shock pairings in context) (Richmond et al., 1999). Consistent with previous studies using multiple shocks, dorsal hippocampal lesions failed to interfere with contextual fear conditioning. Using the delayed conditioning paradigm, amygdala-dependent cued responses were not affected by these hippocampal manipulations.

One reason the ventral hippocampus is thought to be important for contextual fear conditioning, is due to extensive synaptic connections with the amygdala (Maren and Fanselow, 1995). Indeed, the amygdala is critical for the acquisition of both contextual and cued fear conditioning. However, the contribution of the amygdala is region specific. The amygdala consists of several nuclei including the lateral (LA), basolateral (BL), accessory or basomedial (AB or BM), central (CE), medial (ME), and cortical. The roles of these nuclei in fear conditioning have been recently reviewed in detail (Pape and Pare, 2010; Johansen et al., 2011). Lesions of the basolateral amygdaloid complex (BLA) (which includes the LA, BL, and AB nuclei) severely impair both contextual and cued fear conditioning; however, as is the case with the hippocampus, strong overtraining can restore acquisition of fear, e.g., (Zimmerman et al., 2007). The same study demonstrated the lesions of the CE of the amygdala result in severe deficits in conditioned fear that appear resistant to overtraining. Furthermore, a functional CE was necessary for successful overtraining in the BLA lesioned animals (Zimmerman et al., 2007). In conclusion, both the lateral and central nuclei of the amygdala are important for fear conditioning.

### Human studies

Functional imaging studies of brain activity during the acquisition and expression of contextual fear reveal a similar dependence on functional hippocampal and amygdala regions. Virtual reality is a powerful tool for generating reproducible and novel contexts and can be combined with functional imaging of the brain. Using passive observation of two virtual reality environments (an airport and a house), foot shocks were delivered during observation of one context (CTX+) and not the other (CTX−), Alvarez et al. used fMRI to demonstrate functional connectivity between hippocampus and amygdala during the acquisition of the association between the CTX+ and the shock (Alvarez et al., 2008). Unfortunately, modern brain imaging techniques lack the resolution necessary to address which nuclei and sub-nuclei of the amygdala are relevant.

A major limitation in human studies has been the lack of retention of contextual fear. Consistent with other tests of spatial memory (see previous sections), a new generation of studies using full immersion, three-dimensional virtual reality environments appears to improve retention of contextual fear in humans (Huff et al., 2010, 2011).

## Impact of aging on contextual fear conditioning

### Rodent models

A recent review concluded that contextual fear conditioning is not as sensitive to aging as other hippocampal-dependent tasks (Kennard and Woodruff-Pak, 2011). A number of studies have reported no age-related deficit in the ability of aged rats to acquire and remember contextual fear when examined immediately (Kudo et al., 2005), 24 h (Houston et al., 1999; Doyere et al., 2000; Bergado et al., 2011), 72 h (Barrientos et al., 2006), or 10–20 days (Oler and Markus, 1998; Houston et al., 1999) after the initial training. Similarly, little or no age difference is observed for acquisition of contextual fear in mice (Gould and Feiro, 2005; Kaczorowski and Disterhoft, 2009; Woodruff-Pak et al., 2011). In cases where age-impaired retention has been reported, training usually involves low intensity shock and few training sessions. For example, age differences were observed 2 h after a single 2 s, 0.75 mA shock in rats (Kudo et al., 2005; Wati et al., 2006) and 24 h after a single 2 s, 0.2 mA (Fukushima et al., 2008) or 1 mA (Peleg et al., 2010) shock in mice. In contrast, when a single 2 s shock of higher intensity (1.5 mA) was employed, no age difference was observed up to 72 h post training (Barrientos et al., 2006). One group consistently finds age-related deficits 48 h after conditioning using two consecutive training blocks (2 s, 0.5 mA shock/episode) (Gemma et al., 2004, 2005; Mesches et al., 2004); however, age-related differences were not observed 10 days after four training episodes of similar intensity (2 s, 0.4 mA shock/episode) (Oler and Markus, 1998) and similar retention is observed up to 20 days post training following multiple training episodes (Houston et al., 1999). In contrast, using a strong 10 trial paradigm (1 s, 1 mA shock/episode), Moyer and Brown found impaired trace conditioning and contextual fear in aged Sprague-Dawley rats (Moyer and Brown, 2006). The results suggest that the level of arousal induced by the shock intensity or the number of shocks is an important component in determining age differences in retention of fear memories (McGaugh, 2006). It is unclear if the deficits in contextual fear conditioning are due to senescence of the hippocampus or an age-related decrease in activity in brain regions that process arousal and modulate hippocampal function (i.e., the amygdala). Strain differences in sensory decline and age-related neuron loss in relevant brain regions could also play a role in the diversity of results using contextual fear conditioning. Careful attention to these parameters could resolve the apparently contradictory findings.

### Human studies

Contextual fear has not been studied in aged humans. However, when older and younger adults are presented with pictures of fearful faces, both groups activated the same areas, the amygdala bilaterally and the right hippocampus, during successful encoding. Further, activity in the right amygdala and hippocampus was increased in the younger group suggesting that the interaction of these two regions may influence fear responses (Fischer et al., 2010). In fact, several studies have confirmed an age-related decrease in amygdala activation during the processing of emotional or arousing stimuli (Gunning-Dixon et al., 2003; Kaszniak and Menchola, 2012; Moriguchi et al., 2011). Because the amygdala is crucial for the acquisition of both cued and contextual fear conditioning, reduced amygdala activation in the aged population might confound detection of hippocampal-dependent deficits in contextual fear.

### Example rodent protocol

Contextual fear conditioning usually consists of a context and cue as conditioned stimuli to be associated with a strong foot shock as the US. The experiments are conducted within an isolation chamber to control and stabilize environmental variables, including visual, tactile, olfactory and auditory stimuli of the experimental apparatus. Within the isolation chamber, the conditioning chamber consists of modifiable walls and floor to permit changing of contextual elements. For conditioning, the floor consists of a set of parallel rods connected to a foot shock controller for the delivery of shocks of controlled intensity and duration. To change the context, the floor can be replaced with a square grid or a flat surface. The modifiable walls usually include lighting elements as well as a speaker for generating tones to establish both context and cue elements. To change contexts, these elements can be removed, repositioned, or replaced. An additional parameter to establish context is an olfactory stimulus. This is usually provided by placing an odorant (e.g., vanilla or ethanol) in a catch pan under the grid floor.

Fear can be assessed in numerous ways, but all are based on the manifestation of fear as freezing behavior. When a mouse or rat is fearful, they remain motionless for several seconds. The two most common methods for detecting fear include video analysis of behavior (e.g., Coulbourn Freeze Frame) or monitoring movement using infrared lights and detectors or a motion-sensitive floor element (e.g., Med Associates). It is also possible to use telemetry to monitor physiological properties such as skin conductance, heart rate and/or blood pressure.

A typical experiment consists of a 3 day protocol, with two contexts A and B. Context A consists of the foot shock floor, a vanilla odorant, a constant 75 dB 2 kHz hiss (auditory context), chamber light on, and a black wall of the conditioning chamber. Context B consists of a square grid floor, ethanol as an odorant, no hiss, chamber light off, and a white wall of the conditioning chamber.

#### Day 1: fear acquisition

Each animal is transported via its home cage to the experimental room 1 h before experimentation. The animal is placed in the conditioning chamber in context A and behavior is monitored. After 60 s, a pure tone 2.9 kHz is played for 30 s. During the last second of the tone, a 1 s, 1 mA foot shock is delivered. This 90 s training paradigm is repeated for a total of three shocks. One minute after the final shock, the animal is returned to its home cage and housing location.

#### Day 2: context

To test if the animal acquired fear of the context, the animal is transported via its home cage to the experimental room 1 h before experimentation, exactly as on Day 1. The animal is placed in the conditioning chamber in context A and behavior is monitored for 3 min. No foot shock or cue is delivered. The animal is returned to its home cage and housing location.

#### Day 3: cue

Context B is assembled prior to transporting the animal. To test the acquisition of the association of the auditory cue with the foot shock, the animal is transferred to an alternate transport cage (e.g., an opaque box) and acclimated to the experimental room for 1 h prior to experiment. If possible this should be a different location in the room from context A. The lighting of the room can be varied to reinforce the change in context. The animal is placed in Context B for 3 min. After 1 min, the cue (2.9 kHz pure tone) is played. No foot shock is delivered. At the end of acquisition the animal is returned to its home cage and housing location.

### Analysis

Analysis of freezing behavior is usually expressed as the percentage of time spent freezing. Freezing is quantified by setting a minimum duration the animal is still, e.g., 1 s binning, plus a threshold for freezing defined by each animal's behavior. One major advantage of conditioned fear is that freezing behavior is usually very clearly distinguished from normal behavior. Commercial and custom software packages are widely available for standardizing this analysis. Acquisition of contextual fear is assessed by comparing the amount of time spent freezing in context A on day 2 with baseline freezing prior to conditioning on day 1. Cued fear is analyzed by comparing the amount of time spent freezing during the baseline period of day 3 with the time spent freezing during the tone (both in context B).

### Interpretation

Traditionally, context and cue are simply associated with hippocampal and amygdala function. For example, a failure to acquire context despite acquisition of the cue (day 2 vs. day 3) could indicate hippocampal dysfunction. More recently, this clear distinction has been questioned and experimental details can give rise to acquisition of context and/or cue in the absence of hippocampal or amygdala function (reviewed in Maren, 2008). For example, stronger foot shocks or additional acquisition trials can override deficits, presumably by recruiting additional brain regions. The possibility that additional brain regions may contribute or even compensate for age-related impairments in hippocampal and amygdala function reduces the utility of this task for examination of systems level changes due to aging. Optimization of experimental design by using the minimum necessary shock intensity and number, as well as careful attention to age-related sensory deficits, has the potential to overcome these limitations.

### Gaps in linking human and animal laboratory models: future directions

Treatments to alleviate age-related deficits in memory function should be thoroughly tested in animal models. In this regard, interventions should be tested in animal models that reproduce the severity or temporal progression observed for humans. For example, if a treatment is designed to ameliorate cognitive decline, the animal model should demonstrate impairments prior to treatment. If a treatment is designed to prevent cognitive decline, the treatment should be delivered to the animal model prior to the onset of cognitive decline and followed for a period in which impairments normally develop.

In order to prevent/treat age-related cognitive decline, it will be important to develop tasks for the early detection of impaired spatial memory in humans and animal models. In addition, it will be important to track changes in cognitive function over the course of aging and in response to treatments. Work in rodents indicates significant carry-over effects when animals are tested on the water maze at younger and again at older ages (Vallee et al., 1999; van Groen et al., 2002; Vicens et al., 2002; Hansalik et al., 2006). Despite carry-over effects, reliable differences in performance can be observed, beginning in middle-age when animals are trained on a repeated acquisition version of the water maze. The specificity and reliability of repeated acquisition training indicates that this version of the task may be employed for examining the effects of treatments on the ability to rapidly acquire spatial information. However, the reliability as well as carry-over effects associated with repeated testing in humans remains to be determined.

In addition, it will be important to develop measures and procedures that can differentiate normal aging from age-related diseases (e.g., Alzheimer's disease). Virtual reality procedures have been employed in an attempt to differentiate normal aging from Alzheimer's disease (AD). Subjects with AD exhibited enhanced impairment of recognition memory (Zakzanis et al., 2009) and verbal recall of virtual reality environments (Cushman et al., 2008; Widmann et al., 2012). Including a delay can increase the age sensitivity for a number of behavioral tests (Foster, 1999). Middle-aged humans exhibit a decrement in performance for delayed recall of episodic memory (Albert, 1997). Delayed recognition span tests have also been employed to show that middle-aged monkeys exhibit impairments specifically for retention of spatial information (Herndon et al., 1997; Moss et al., 1997; Lacreuse et al., 2002). In rats, no age-related deficit is observed for fear conditioning to a tone when the CS and US overlap in time; however, introduction of a delay interval between the CS and US (i.e., trace conditioning; Figure 4) revealed age-related decrements in the acquisition of freezing behavior (Moyer and Brown, 2006). Delay-dependent memory deficits have been well-characterized on the water maze. An age-related impairment in spatial memory is observed 24 h after a single training session (Foster et al., 1991, 2001, 2003; Foster, 1999; Norris and Foster, 1999; Foster and Kumar, 2007) or on the first trial each day during training across days (Gage et al., 1984; Rapp et al., 1987; Aitken and Meaney, 1989; Foster, 2012a) (Figure 3). Another version of the water maze, delayed matching-to-sample, involves changing the platform location each day and performance is examined over two trials. Normally, animals exhibit a decrease in the latency or distance to find the platform on the second trial. This decrease or savings is lost with advancing age as the inter-trial interval is increased. Thus, young rats exhibit retention for up to 6 h; middle-aged animals begin to exhibit retention deficits as the delay is increased to 2 h and aged animals exhibit deficits for delays greater than ~1 h (Means and Kennard, 1991; Bizon et al., 2009). While two trial versions of the spatial maze have not been examined during normal aging in humans, on a human analogs of the water maze, memory declines with increasing memory load and delay interval in younger humans (Fitting et al., 2007).

In conclusion, hippocampal-dependent spatial learning and memory are particularly vulnerable to age-related decline in humans and animals models of aging. Virtual environment technology permits the use of procedures employed in animal models in order to translate findings across different levels of analysis. Furthermore, the tasks can be designed to focus on different aspects of learning and memory in order to increase the sensitivity of the tasks to individual differences in the emergence of cognitive decline and point to mechanisms for declining memory function. It is important to control for age-differences in sensory-motor function, previous experience, and the level of arousal evoked by the task. Continued development will be required to insure that behavioral tasks can track changes in cognitive function over the course of aging and in response to treatments.

### Conflict of interest statement

The authors declare that the research was conducted in the absence of any commercial or financial relationships that could be construed as a potential conflict of interest.
